# A Universal and Efficient Detection of Chytridiomycosis Infections in Amphibians Using Novel Quantitative PCR Markers

**DOI:** 10.1155/2023/9980566

**Published:** 2023-04-30

**Authors:** Gayathri Sreedharan, Yashwant Singh Panwar, Saketh Murthy, Kaya Klop-Toker, Roberto Ibáñez, Estefany E. Illueca, Rebecca Webb, Venu Govindappa, Barkha Subba, Harika Segu, Krishna Pavan Kumar Komanduri, Karthikeyan Vasudevan

**Affiliations:** ^1^CSIR-Centre for Cellular and Molecular Biology, Hyderabad, India; ^2^School of Environmental and Life Sciences, University of Newcastle, Australia; ^3^Smithsonian Tropical Research Institute, Panama City, Panama; ^4^Sistema Nacional de Investigación, SENACYT, Panama City, Panama; ^5^College of Public Health, Medical and Veterinary Sciences, James Cook University, Townsville, Queensland, Australia; ^6^Department of Zoology, Centre for Applied Genetics, Jnana Bharathi Campus, Bangalore University, Bengaluru, Karnataka, India; ^7^Department of Biosciences, Chandigarh University, Mohali, Punjab, India; ^8^Padmaja Naidu Himalayan Zoological Park, Darjeeling, India; ^9^Ashoka University, Rajiv Gandhi Education City, Sonipat, Haryana, India

## Abstract

Chytridiomycosis is an infectious disease in amphibians caused by two chytrid fungi, *Batrachochytrium dendrobatidis* (*Bd*) and *Batrachochytrium salamandrivorans* (*Bsal*), and is the worst infectious disease known in wildlife so far. Worldwide spread of the disease has caused unprecedented loss of global amphibian diversity. Although some lineages of *Bd* are enzootic and are not as deadly as the pandemic lineage, nearly 40% of amphibian species are still declining globally due to chytridiomycosis. Efficient surveillance and monitoring of chytridiomycosis are the immediate safeguard against rapid declines or extinctions of amphibian populations. Previous studies showed that existing diagnostic assays were not sensitive to certain *Bd* haplotypes like those from Korea, China, India, Japan, and Brazil and thereby, there is a need for a universal, sensitive, specific, reproducible, and affordable diagnostic assay. We designed a one-step SYBR green-based quantitative polymerase chain reaction (nSYBR qPCR) for robust detection of *Bd.* It amplifies an 82 base-pair segment between the 5.8S rRNA and ITS2 of the *Bd* genome. The primer pair was tested *in-silico* on 40 isolates from four known *Bd* lineages. Using skin swab samples of wild amphibians and cultured zoospores from Australia and Panama, we compared the clinical specificity and sensitivity of the newly described primers to the existing TaqMan-based qPCR assay. From India, we used samples which had been previously tested with Nested PCR to validate the new primer pairs. The newly described primer pair was then tested on swab samples from Anura, Caudata and Gymnophiona from India. We report widespread chytridiomycosis with varying infection loads on them. The new assay showed comparable efficiency to the TaqMan-based qPCR assay. This diagnostic assay can facilitate widespread surveillance of chytridiomycosis where it has been previously absent, which may reveal several reservoirs of the pathogen and can improve our understanding of this important wildlife disease.

## 1. Introduction

Chytridiomycosis is an emerging infectious disease (EID) caused by two species of pathogenic fungi, *Batrachochytrium dendrobatidis (Bd)* and *Batrachochytrium salamandrivorans* (*Bsal*). It has resulted in catastrophic declines of over 500 amphibian species and extinctions of 90 amphibian species worldwide [1]. Four important lineages of *Bd* are recognized, namely, *Bd*ASIA-1 (Asia), *Bd*ASIA-2/*Bd*Brazil (Brazil), *Bd*CAPE (Europe and Africa), and *Bd*GPL (global) [2]. All the lineages, except *Bd*GPL, are enzootic and restricted to specific geographical locations [3]. Initially, geographic regions with high levels of *Bd* infection and frog mortality were the major focus of research, and then areas with enzootic *Bd* have been recognized. Particularly, from Asia, such areas are yet to be identified [4]. Korea, China, Japan, and India are among the countries where *Bd* is enzootic [5–10].

Rapid detection of chytridiomycosis is essential in managing and mitigating the disease [11]. Polymerase chain reaction (PCR)-based tests are routinely used to diagnose the disease [7, 12, 13]. The gold standard for *Bd* diagnosis has been a TaqMan-based quantitative PCR [13]. However, this qPCR method does not detect Asian haplotypes [7, 8]. For this, a nested PCR is an alternative, but it is an agarose gel-based assay, and it does not provide the infection load. For epidemiological studies, it is essential to know the infection status of the host and also a load of infection on the host [14]. Therefore, any compromise on sensitivity or specificity would impact the outcomes of such studies.

In the Indian subcontinent, *Bd* infections have no clinical symptoms and occur with low prevalence and high haplotype diversity [4, 8]. The conventional TaqMan-based qPCR assay, hereafter, referred to as Boyle's assay, showed low sensitivity because *Bd* in the region had insertion-deletion and substitution of bases in the probe-binding and reverse primer-binding sites [8]. We aimed to develop a reproducible, sensitive, and specific assay for *Bd* detection in amphibians. We designed a SYBR-green-basedone-step qPCR primer set. For this, we first searched for a region in the genome of *Bd* that: (a) is accessible in databases, (b) has multiple copies in the genome, and (c) has the least number of mutations. The internal transcribed spacer (ITS) region of *Bd* fits these criteria and we used ITS sequences deposited in NCBI with partial ITS1, 5.8SrRNA, and partial ITS2 segments. Then, we used this ITS sequence assembly to screen for potential primer sites for amplification of *Bd* and validated the primers. The primers reliably amplified *Bd* lineages in both *in-vitro* and *in-silico* PCRs. We report the efficiency of the quantitative PCR primer pair on field samples from India, Australia, and Panama. The primer pair is sensitive, specific, and reproducible for different geographic areas and lineages of *Bd* (BdASIA-1, *Bd*ASIA-2/*Bd*Brazil, *Bd*CAPE, and *Bd*GPL). Using the new primer pair, we report widespread *Bd* on Caudata and Gymnophiona in India and also higher infection prevalence of *Bd* in amphibians than those reported previously.

## 2. Materials and Methods

### 2.1. Designing of Primers

To produce a universal *Bd* primer set, we used Primer BLAST, which combines BLAST with a global alignment algorithm to ensure complete primer-target alignment while eliminating primers with many mismatches with the target [15]. We used the ITS FASTA sequence available in NCBI, JEL197 (*Bd*GPL) (AccessionID:NR_119535.1) as the template for primer design, and the primer search parameters avoided regions with single nucleotide polymorphisms (SNPs) and restricted the number of mismatches in primers. We matched the potential primer pairs with an alignment (made using MEGA version X) of 440 partial ITS1-5.8S-partial-ITS2 sequences of *Bd* from NCBI [16]. These 440 sequences were deposited from different continents and were a good representative of the global *Bd* ITS diversity. We eliminated those primer pairs that had multiple mutations when aligned with the global *Bd* ITS dataset mentioned above. We used the primer pairs that qualified this criterion for further analysis using Primer BLAST. We performed BLAST using these primers on a query set that excludes *Bd* (Taxid: 109871 by clicking on the exclude dataset option from “Choose Search set” in BLAST window). This step ensured that no off-target amplifications occurred with the primer pair. The chosen primer set did not match off-target templates, and we proceeded with downstream *in-vitro* standardization. ChFP1-5′ CGCACATTGCACTCGTAA 3′ and ChRP1-5′ GGTTCATATCTGTCCAGTCAATTC3′ were the primer pair we used for all our analyses.

To determine the optimal annealing temperature with the lowest dilution (100*x*), we ran a wide temperature gradient from 52°C to 64°C on a 2.5% agarose gel with 10*x* and 100*x* dilutions of the positive control (gblock™ dsDNA of the whole ITS reference (JEL197, 476 bp) with a stock solution concentration of 200 ng/*µ*l). We performed nSYBR qPCR with the new primer pairs *in-vitro* on (i) field amphibian swab samples from India (Anura, Caudata and Gymnophiona), (ii) swab samples from wild anurans in Australia and cultured zoospores (*Bd*GPL strain JEL423), and (iii) swab samples from wild anurans in Panama and cultured zoospores (*Bd*GPL strain JEL423)). This was to ensure amplification in different labs on various types of swab samples from different species of amphibians. Since we did not have access to cultured zoospores or swab/tissue samples from other lineages, we also validated the new primer pairs *in-silico* for their ability to amplify *Bd* sequences by extracting ITS regions from their raw reads deposited in the NCBI SRR database [2]. We chose a set of 40 isolates from Africa, Asia, South America, and Europe. These accessions included all the known lineages of *Bd* (Supplementary Table S1) and one hybrid isolate. We screened the consensus ITS regions of these samples for mutations at the primer-binding regions (Supplementary information S1).

### 2.2. Bioinformatics

O'Hanlon et al. [2] generated DNA sequences from 216 isolates of *Bd* from across the globe and deposited the raw reads in NCBI. We chose 40 isolates representing various geographical locations and assembled their ITS regions (Supplementary Table S1). The SRR files included all known lineages of *Bd* from the NCBI SRR database (NCBI Bio Project accession PRJNA413876). These were aligned to the *Bd* reference genome from NCBI (JAM81 v1.0, Accession ID: NW_006281099.1) using HISAT2 with default parameters [17]. After performing the alignment of the reads, we performed variant calling using bcftools mpileup. Finally, the consensus whole genome sequences were called from the vcf output using bcftools. Using BLAST, the consensus sequences of the ITS regions were identified on the reference ITS sequence from NCBI (JEL197 ITS region, Accession ID: NR_119535.1). We screened the sequences for mutations in the primer-binding regions. The GATK pipeline for variant calling was also used on a subset of the isolates, resulting in the same results as bcftools mpileup. We set all command sat default parameters; all the alignments had sufficient read depth and per-base coverage. A detailed pipeline of analyses used is in Supplementary information S1.

### 2.3. Study Sites and Samples

This study was validated using 227 anuran skin swabs from Tillari Conservation Reserve (TCR) collected between 2014 and 2016 (*n* = 137) and 2018–2021 (*n* = 90). Results from the nSYBR qPCR were compared with those obtained from an earlier study using nested PCR on the same samples (*n* = 137) from the study site [8]. In addition to this, we used newly collected swabs, including 57 caecilian (Gymnophiona) skin swabs from Bangalore University, Karnataka, India collected on 5^th^ March, 2021; and 11 salamander (Caudata) swab samples from Padmaja Naidu Himalayan Zoological Park (PNHZP), Darjeeling, India in December 2020. To check the ability of the new primer pairs to amplify *Bd* from other geographical locations, we also tested our new primers for *Bd* on wild-caught anurans and cultured zoo spores (*Bd* JEL423) from Australia (*n* = 60) and Panama (*n* = 43). All anurans in the field in India were caught between 18:00 to 24:00 hrs with separate nitrile gloves and new plastic bags to prevent cross-contamination between samples. The same protocol was used to collect and swab both salamander and caecilian samples. We swabbed the skin of each frog 70 times using sterile cotton swabs (HIMEDIA®PW003) following the protocol by Kriger et al., [18]. We obtained Institutional Animal Ethics Committee approval for animal handling (IAEC19/2018) and permission from the Maharashtra State Forest Department to collect samples from TCR (Desk-22(8)/WL/CR-4(18-19)/322/2018-2019).

### 2.4. DNA Extraction and PCR Conditions

DNA was extracted from the cotton swabs using the protocol from Goka et al., [7] with minor modifications [8]. Cotton swabs were excised and placed in 2 ml tubes containing 400 *µ*l of lysis buffer (1 mg/ml Proteinase K, 0.01 M NaCl, 0.1 M EDTA, 0.01 M Tris-HCl of pH 8.0, 0.5% Nonidet P-40) [7] and three tungsten carbide beads of 3 mm diameter each. These were homogenized in Qiagen Tissue Lyser II for 2 min and centrifuged at 8,000 rpm for 2 min. The supernatant with DNA was then transferred to fresh 1.5 ml tubes and incubated at 50°C for two hours, followed by incubation at 95°C for 20 min. After incubation, we centrifuged the samples at 13,000 rpm for 10 min. The supernatant was transferred into fresh 1.5 ml tubes and stored at −20°C till required for PCR. We diluted the DNA extract with nuclease-free water toa final dilution of 1 : 100. This was the template for our qPCR assays DNA extraction from swab samples from amphibians in Australia and Panamania followed the procedure outlined in Kriger et al., [18].

We used the Roche Light cycler® 480-II real-time PCR machine for our experiments. A reaction volume of 15 *µ*l contained 1X Takara master mix (TB Green Premix Ex Taq II, RR820 A), 200 nM of forward and reverse primer, 1X ROX (TAKARA Bio Inc.), 5 *µ*l of diluted DNA, and the remaining volume made up by nuclease-free water. Cycle conditions for qPCR were as follows: 95°C for 30seconds for initial denaturation at 4.40°C/sec ramp rate, 95°C for 5 secs (ramp rate: 4.4°C/sec) and 58°C for 30 seconds (ramp rate: 2.2°C/sec) for 40 cycles, and a final melt curve program of 95°C (hold: 5 secs, ramp rate: 4.4°C/sec), 58°C (hold: 1 min, ramp rate: 2.2°C/sec) and 95°C (continuous acquisition mode, ramp rate: 0.11°C/sec, and 5 acquisitions/sec). We carried out all reactions in triplicates with positive (*Bd* JEL197 or JEL423 DNA standard) and negative control (nuclease-free water). The qPCR amplicons were loaded on a 2.5% agarose gel to check for additional peaks in the melt curve program. The additional band that did not correspond to the size of the positive control in our sample was eluted using QIA quick Gel Extraction Kit and ligated into pJET1.2/blunt vector using Clone JET PCR cloning kit. Transformed *E. coli* DH5*α* cells were selected. We selected the transformed colonies against Ampicillin resistance on LB Agar plates. Colony PCR with the *Bd*-specific primers confirmed transformation. The plasmid was isolated from the transformants using the alkaline lysis method [19]. These were later amplified using the pJET1.2 forward and reverse primers and sequenced in triplicate. These sequences were BLASTed with VecScreen to eliminate the vector sequences. The remaining sequence was, in turn, BLASTed to see if they were indeed targeted amplification. We also sequenced positive samples to check if they were the desired amplicons.

### 2.5. Efficiency of qPCR Markers, Analytical Sensitivity and Specificity

We used synthesized gblock™ dsDNA of the whole ITS reference (JEL197, 476 bp) as a template to amplify an 82 bp long stretch (region including partial 5.8S and partial ITS2 from the reference) using conventional PCR with the newly designed primers. We eluted the amplicon using QIA quick Gel Extraction Kit. We quantified the DNA concentration in ng/*µ*L using NanoDrop™ Spectrophotometer. We calculated the amplicon copy numbers present per *µ*L by taking an average base pair weight of 650D and Avogadro's number (6.022 × 10^23^). The following equation was used: copy number = (DNA (in ng) × (6.022 × 10^23^ molecules/mol))/(length of DNA in base pairs × 1 × 10^9^ ng/g × 650 g/mol). We performed a 10X dilution from this copy number (in our case, 10^8^ copies) up to 1 copy per *µ*L and used these as standards to quantify copy numbers in this qPCR assay. We plotted the quantification cycle number (Cq) against the logarithm of the DNA copy numbers of the standards used. The efficiency was calculated from a linear regression of this plot as (10^(−1/slope)^ − 1)*∗*100. We used regression to compute R^2^ in program R's Base R package [20].

We serially diluted rDNA of the ITS region of *Bd* JEL270 (constructed at Pisces Molecular) to construct a standard curve for assays run with Australian samples. We constructed standard curves using cultured zoospores of JEL423 for analyzing wild frog skin swab samples from Panama by making 10X dilutions from 10,000 zoospore equivalents (ZE) to 0.1 ZE.

We calculated analytical sensitivity and specificity using a Limit of Detection/Limit of Quantification (LoD/LoQ) Assay. In this method, we got both the discrete LoD/LoQ thresholds that represented the lowest standard that amplifies 95% of the times (LoD) and the lowest standard that accurately amplifies the copies of DNA with a coefficient of variance less than 35% in all its replicates [21]. Besides this, the assay also uses a curve-fitting method to derive LoD/LoQ for the assay. To build the curve, we made 23 replicates of 4X dilutions ranging from 1024 copies to 1 copy per *µ*L along with standards ranging from 10^8^ copies per reaction to 1 copy per reaction. We used R packages *drc* and *ggplot2* to derive the standard curve plots, a plot of the LoD and LoQ models. We also included no template controls (NTC) in this plate [21]. We checked the melt curve plots after the run to ensure that there were no nonspecific amplicons in the standards. We did not use the dataset if there were nonspecific amplifications. We used the cut-off (Cq = 33) from this assay for all analyses, using ITS copy numbers to construct standards. When zoospore equivalents were used to build standards, we considered all samples positive that amplified within 35 cycles of qPCR. The experiments to run standards and all the validation assays performed with different samples were all carried out in triplicates.

### 2.6. Clinical Sensitivity, Specificity and Relative Efficiency

Clinical sensitivity and specificity were measured relative to the existing gold standard of *Bd* diagnosis: TaqMan probe-based qPCR in samples from Australia and Panama. From Australia, we used 60 samples (44 swab samples from wild-caught *Litoria littlejohnii* and 16 isolated chytrid samples of varying dilutions) to validate our one-step SYBR-green-based assay. We ran 60 samples in triplicate using the TaqMan probe and the SYBR-green assay and compared them using a confusion matrix. From Panama, 43 samples were tested, including 29 known *Bd* positive and 14 *Bd* negative samples that included 20 species. From India, we tested our one-step SYBR-green-based assay on 137 samples collected from TCR between 2014 and 2016 that were also tested with nested PCR in an earlier study [8].

To compare the two assays, we used a two-by-two matrix where TaqMan-based qPCR was fixed as the reference assay and compared with the new SYBR-green-based qPCR (index assay) [22]. From this diagnostic, sensitivity and specificity were calculated as *estimated sensitivity* *=* *100%* × *True Positive*/(*True Positive* *+* *False Negative*); *estimated specificity* *=* *100%* × *True Negative*/(*False Positive* *+* *True Negative*). We computed their 95% CI using the modified Wald method. We calculated relative efficiency as a trade-off between precision and cost. When the two methods had relatively similar accuracy, precision was compared for a fixed cost. Using a linear cost function, which included an initial setup cost (sample procurement, DNA isolation, instrument, and plates) *c*_0_, cost *c*_1_, and *c*_2_ for TaqMan and nSYBR experiments, respectively; initial sample size for TaqMan as *n*_1_ (equation (1)); *n*_*c*_ as the cost corrected sample size for nSYBR (equation (2)).

(1) $$\begin{array}{c} c_{T}=c_{0}+c_{2}n_{1}\text{,} \end{array}$$

(2) $$\begin{array}{c} c_{T}=c_{0}+c_{1}n_{c}\text{.} \end{array}$$

We used the analyses cost as 2.5 USD/sample and 5.5USD/sample for nSYBR and TaqMan-based methods, respectively [23, 24]. The initial setup cost *c*_0_ is the same for both methods, so we arrived at a total cost of 65 USD for 11 TaqMan samples.

We solved equation (2) and calculated nc as 25. Using bootstrapping function in Program R, we resampled 1000 times 25 *Bd* load values from SYBR-green and 11 from TaqMan. We estimated the standard error (*σ*) and 95% confidence limits (upper and lower bound of *σ*) of the *Bd* load measured using the two methods after adjusting the sample size for a fixed cost. We took the ratio of the upper and lower CI values of as the bounds of the relative efficiency of the assay. This value should be close to 1. If the relative efficiencies are similar, it will be <1 when TaqMan is more efficient than nSYBR and >1 when nSYBR is more efficient than TaqMan.

For samples from India, our reference assay was Nested PCR, and the test assay was nSYBR with new primers, so we calculated the prevalence for the two assays and compared the 95% CI. We used the Wilson Score interval method with the Hmisc package Program R to estimate the 95% confidence interval of the proportions. We also compared the ITS copy numbers/zoospore equivalents reported from the assays using a one-way analysis of means (not assuming equal variances) and a two-sample *t*-test in Base R package.

## 3. Results

### 3.1. qPCR Primer

**Figure 1:**
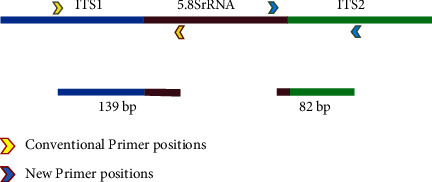
A schematic representation of the positions within the partial ITS1- 5.8SrRNA-ITS2 that the new primers amplify, the length of the amplicon relative to the position and size of the conventional amplicon.

The new primer pair amplified an 82 base pair (bp) region between 5.8S and ITS2. The existing TaqMan probe-based primers amplified the 139 bp region between ITS1 and 5.8S (Figure 1). After the temperature gradient PCR, we arrived at 58°C as the optimum temperature, as it did not produce multiple bands and amplified well at low dilutions (Supplementary Figure S1). The consensus ITS sequences from 40 *Bd* isolates did not have any mutations in the primer-binding regions (Supplementary Figure S2). It was validated using the primer pairs ChFP1 and ChRP1 to diagnose *Bd* across all geographic regions and lineages.

### 3.2. qPCR Efficiency

**Figure 2:**
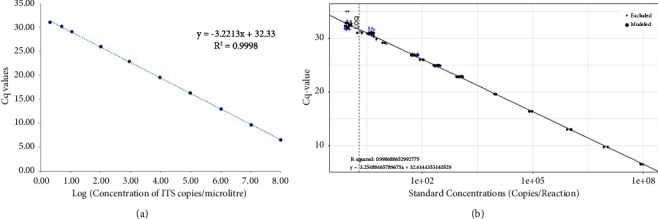
(a) Standard curve constructed for dilutions of ITS copy numbers from 10^8^ copies per reaction to 1 copy/reaction (b) An LoD-LoQ Assay performed using 23 replicates each of lower concentration of 1024, 256, 64, 16, 4 and 1 ITS copies per reaction, along with standards starting from 10^8^copies per reaction to 1 copy per reaction (Figure 2(a)) to calculate the lowest standard that can be detected with 95% confidence (LoD) and the lowest quantity that can be amplified with less than 35% covariance.

Amplicon size for the primer pair used on samples was 82 bp. It matched with the output from Primer BLAST and the amplicons from positive standards in the qPCR (Supplementary Figure S3A). The melting temperature (Tm) of all the positive samples and positive standards was 77°C (SD = 0.26) (Supplementary Figure S3B). In some cases, there was an additional Tm at 79°C when fresh dilutions of DNA from swab samples were not used (Supplementary Figure S3C). The product was 65–70 bp in size (Supplementary Figure S3A), and its sequence matched with a partial *Bd* sequence from Hungary (Accession ID: MH745069). We calculated qPCR efficiency as 100.9% with an *R*^2^ = 0.99 (Figure 2(a)). LoD was fixed at one copy/reaction using the 23 intraassay replicates. LoQs were at two copies with a coefficient of variation of below 35%. We made the cut-off as 33 cycles based on these values (Figure 2(b)). Samples amplified after 33 cycles were considered negative.

### 3.3. Sensitivity, Specificity and Relative Efficiency

The confusion matrix produced percentages of accuracy, misclassification, sensitivity, and specificity (Table1. We report an accuracy of 0.90 (95% CI = 0.79–0.95), misclassification of 0.10 (95% CI = 0.04–0.20), sensitivity of 0.89 (95% = CI 0.73–0.96), and specificity of 0.92 (95% CI = 0.74–0.99). ∆Cq represented differences in the Cq value between the two assays (Supplementary Figure S4). The Cq value from the new primers was lower than those of the conventional primers (∆Cq ± *sd* = 5.71 ± 3.00). It implied amplicon production earlier for the new primers than the conventional ones. This means that our assay was slightly more sensitive than the existing method. The ITS copy numbers reported by both assays, however, were not different from each other (*F* = 2.1774, *p* = 0.1465, Supplementary Figure S5A). Based on zoospore equivalents of *Bd* from Panama, a comparison between the two assays resulted in the same identities of samples as positives (Supplementary Table S3). However, there was a significant difference in the zoospore equivalence for the assays performed on samples from Panama (*t* = 2.344, d*f* = 56, *p*-value = 0.0227, Supplementary Figure S5B). We found the relative efficiency of the new assay (0.834; 95% CI: 0.821–0.847) to be slightly lower in comparison to the conventional assay.

### 3.4. Status of *Bd* in Asian Amphibians

We tested samples from different geographical locations belonging to different orders of Amphibia using the new primers. Salamanders (*Tylototriton himalayanus*) were positive for all the 11 samples tested with high infection loads (Table 2, Supplementary Table S2). We compared nested PCR and SYBR-green-based qPCR results on the field samples from anurans in TCR. With nested PCR, the prevalence was 1.4% (95% CI = 0.1–5.4), and with the new primers in nSYBR, the prevalence was 70.9% (95% CI = 64.5–76.7) for the same samples (Supplementary Table S). Forty-four of the 57 caecilian samples were positive for *Bd* infection with high infection loads (Supplementary Table S2).

## 4. Discussion

The new primer set developed improved detection and sensitivity and comparably quantified the *Bd* load with the TaqMan primers. The results were reproducible as different labs produced repeatable results based on the assay and reliably amplified different *Bd* lineages. Importantly, this one-step PCR primer pair was able to amplify *Bd* strains, which could not have been amplified with the available markers. Such rapid methods are always preferred as the source of DNA is from a noninvasive sample, which might have compromised DNA quality [25]. Furthermore, the short amplicon length reduces the probability of PCR failure from low-quality DNA samples [26]. Consequently, our qPCR efficiency was 90–110%, which is generally considered acceptable [27, 28]. Melting temperature (*T*_m_) was consistent in all the positive samples at 76-77°C, showing that only the desired amplicon was produced. This pattern was consistent with field samples from India and across the three labs. Therefore, it is a specific and sensitive assay that shows reproducible patterns of detecting *Bd* in different labs. It could serve as a diagnostic tool for the detection of *Bd* globally.

Caecilians from Asia are known to have *Bd* [29]. This study reported high prevalence based on 19 swab samples from 4 species of caecilians. We tested 57 individuals belonging to 5 species and detected high ITS copies in them, suggesting that caecilians could be reservoirs in the spread of *Bd* (see Supplementary Table S2). For the first time, we also report *Bd* infection from a captive population from Himalayan salamanders (*Tylototriton verrucosus*) in India. Out of the 11 salamanders tested, all were positive with a high average infection load (Supplementary Table S2). The Himalayan salamander is a near-threatened species, and the adult population faces multiple anthropogenic pressures [30]. This study highlights the importance of monitoring the disease burden in a community of amphibians that share the same habitats as larvae and adults. However, amphibian community-level assessment of *Bd* infection status globally would be possible only when cost-effective and efficient alternatives such as the new nSYBR assay are available.

Internal Transcribed Spacer region contains three partitions as follows: ITS1, 5.8S, and ITS2. The 5.8S and flanking regions are conserved and contain a sufficient number of informative sites for plants and fungi. There are multiple copies of this in the genomes of all organisms. It makes this target to be desirable for designing primers, as the multicopy nature of ITS lends testing sensitivity to the diagnostic assay [31]. However, biases in the estimates of copy numbers might be due to the differences in copies of ITS in the reference genome or number of *Bd* zoospores used for constructing standard curves and the copies of ITS in the target *Bd* strain. When the TaqMan-based assay was designed, the ITS copies in a single zoospore were kept at 10 [13]. Since then, chytridiomycosis research has revealed a complex and dynamically evolving *Bd* genome. Multiple *Bd* lineages have ITS copies that range from 10 to 169 per zoospore [32, 33]. There was a significant difference in the copy numbers of *Bd* reported using assays in samples from Panama (Supplementary Figure S5B). This might be because the standards were constructed using reference genomes that might have a high copy number of ITS [31]. Another explanation could be that intragenomic sequence heterogeneity in copies of ITS, which has been reported from other pathogenic fungi, could also be present in *Bd* and might have caused this difference [34]. If mutations were in the forward primer, reverse primer, or probe-binding regions, the TaqMan-based assay would not detect such copies. It could have also led to a large standard error associated with the estimate of infection loads by the nSYBR assay. Accurate estimations of the pathogen load using noninvasive diagnostic procedures are not always feasible [14]. We suggest categorizing the data into low, medium, or high infection burden categories for analysis.

Previous studies have estimated step-wise costs of processing a sample from the collection of swabs and DNA isolation to qPCR [23, 24]. Using those cost estimates, we arrived at the per sample cost of the two methods and calculated the relative efficiency of the assays. The new nSYBR assay showed comparable efficiency for the same fixed costs as the TaqMan assay. TaqMan-based assay is well-known for its specificity when compared to a SYBR-green-based assay. For short amplicons (<100 bp), it is tedious to design a TaqMan probe. We recognize this is a distinctive research problem that needs to be addressed in the future.

Cold spots are areas with high infection prevalence with no visible effects on the host populations [4, 35]. Susceptible, resistant, and tolerant hosts are the recognized categories in an amphibian community burdened with *Bd* [36]. Therefore, widespread testing at the amphibian community level is a prerequisite to assessing infection status in host populations [4, 35]. *Bd* has caused historical declines in amphibian communities globally, and our understanding of this pathogenic fungus has changed dramatically over the last decade. We expect that many *Bd* reservoirs will be identified through monitoring programs involving efficient diagnostic assays. In the same light, host-pathogen coevolution and the pathways of transition of the pathogen from enzootic to epizootic or *vice versa* could also be effectively studied [35]. As new strains of *Bd* are evolving in different geographical areas and spreading through unabated global trade of amphibians, large-scale surveillance for new emergent virulent/hybrid strains of *Bd* is necessary [3, 37]. The new SYBR assay would help in facilitating this globally, including detection of emerging hybrid strains.

## Figures and Tables

**Table 1:** Comparison of TaqMan-based qPCR assay using Boyle's primers and SYBR-green based qPCR using the new primers. Samples that are positive in both the assays were true positive (TP), positive in Boyle's assay and negative in the new assay as false negative (FN), positive for the new assay and negative for Boyle's assay as false positive (FP) and negative for both as true negative (TN).

Boyle's Primer set	New Primer set		
		Positive	Negative
	Positive	31	4
	Negative	2	23

**Table 2:** The number of samples tested and positive samples from different populations of Amphibia.

Country	Source	Number of samples and number of species	Number of positive samples
India	Tillari Conservation Reserve, Maharashtra (*In-situ*)	227 samples from 11 species of Anura	161
	Padmaja Naidu Himalayan Zoological Park (*Ex-situ*)	11 samples from 1 species of Urodela	11
	Bangalore University (*Ex-situ*)	57 samples from 5 species of Caecilians	44

Australia	New South Wales (*In-situ*)	60 samples from 1 species of Anura	33

Panama	Panama (*In-situ*)	43 samples from 20 species of Anura	29

## Data Availability

The data used to support the findings of this study are available from the corresponding author upon reasonable request.

## Supplementary Materials

Supplementary Materials Supplementary table S1 (SRR files of 40 *Bd* isolates (NCBI BioProject accession PRJNA413876) chosen for alignment and reconstruction of ITS sequence, with the SRR and sample IDs, geographical information and identity of strain). Supplementary table S2 (All the samples tested for *Bd* with the SYBR-green-based assay using the new primer set including anuran samples from 2014-2021, caecilian samples from Bangalore University and salamander samples from Darjeeling). Supplementary table S3 (All samples tested at Panama, with species ID, infection status of the tested sample, and the mean Cq and concentration of the *Bd* ZE in the sample). Supplementary figure S1 (The agarose gel picture of a temperature gradient PCR done with the positive control (JEL197) as template. The temperature and the dilution factor of the template is marked). Supplementary figure S2 (Figure shows the forward and reverse primer-binding sequences (shown in red) within the ITS for all the 40 sequences aligned from the raw short read files, covering all the lineages and continents). Supplementary figure S3 ((A) An agarose gel image showing the amplicon at 82 bp of the positive control. The field samples with a second band below the 82 bp amplicon is shown. (B) The melt curve corresponding to the standards constructed with JEL197 at 77°C. (C) The melt curve plot in which some samples have an additional peak at 79-80°C). Supplementary figure S4 (A graph that shows the ∆Cq values and indicates the difference in the amplification cycles of Boyle's and New primers). Supplementary figure S5 ((A) A box plot showing the distribution of the reported ITS copy numbers between Boyle's primers and new primers. These data compare the infection loads of samples (*n* = 31) that are positive for both the methods. (B) The Log_10_ Zoospore Equivalents reported by Boyle's TaqMan-based assay and the new SYBR-green-based assay (*n* = 30). (C) Standard curve for the JEL423 with the new primers using the protocol. (D) Standard curve for JEL423 with Boyle's primers). Supplementary information S1 (Whole genome alignment and variant calling using bcftools and GATK). Supplementary information S2 (MIQE Checklist).
