# Supplementary material for: A Universal and Efficient Detection of Chytridiomycosis Infections in Amphibians Using Novel Quantitative PCR Markers
Source: Transbound Emerg Dis. 2023 Apr 30;2023:9980566. doi: 10.1155/2023/9980566 (PMC12017111; doi:10.1155/2023/9980566)
Supplement: Supplementary Materials — Supplementary table S1 (SRR files of 40 Bd isolates (NCBI BioProject accession PRJNA413876) chosen for alignment and reconstruction of ITS sequence, with the SRR and sample IDs, geographical information and identity of strain). Supplementary table S2 (All the samples tested for Bd with the SYBR-green-based assay using the new primer set including anuran samples from 2014-2021, caecilian samples from Bangalore University and salamander samples from Darjeeling). Supplementary table S3 (All samples tested at Panama, with species ID, infection status of the tested sample, and the mean Cq and concentration of the Bd ZE in the sample). Supplementary figure S1 (The agarose gel picture of a temperature gradient PCR done with the positive control (JEL197) as template. The temperature and the dilution factor of the template is marked). Supplementary figure S2 (Figure shows the forward and reverse primer-binding sequences (shown in red) within the ITS for all the 40 sequences aligned from the raw short read files, covering all the lineages and continents). Supplementary figure S3 ((A) An agarose gel image showing the amplicon at 82 bp of the positive control. The field samples with a second band below the 82 bp amplicon is shown. (B) The melt curve corresponding to the standards constructed with JEL197 at 77°C. (C) The melt curve plot in which some samples have an additional peak at 79-80°C). Supplementary figure S4 (A graph that shows the ∆Cq values and indicates the difference in the amplification cycles of Boyle's and New primers). Supplementary figure S5 ((A) A box plot showing the distribution of the reported ITS copy numbers between Boyle's primers and new primers. These data compare the infection loads of samples (n = 31) that are positive for both the methods. (B) The Log10 Zoospore Equivalents reported by Boyle's TaqMan-based assay and the new SYBR-green-based assay (n = 30). (C) Standard curve for the JEL423 with the new primers using the protocol. (D) Standard curve [file 9980566.f1.docx]

**A universal and efficient detection of chytridiomycosis infections in amphibians using novel quantitative PCR markers – Sreedharan et al.**

Gayathri Sreedharan^1^, Yashwant Singh Panwar^1^, Saketh Murthy^1^, Kaya Klop-Toker^2^, Roberto Ibáñez^3,4^, Estefany E. Illueca^3^, Rebecca Webb^5^, Govindappa Venu^6,%^, Barkha Subba^7^, Harika Segu^1^, Krishna Pavan Kumar Komanduri^1#^, Karthikeyan Vasudevan^1*^

^1^CSIR-Centre for Cellular and Molecular Biology, Hyderabad, India

^2^School of Environmental and Life Sciences, University of Newcastle, Australia

^3^Smithsonian Tropical Research Institute, Apartado, Panama

^4^ Sistema Nacional de Investigación, SENACYT, Apartado, Panama

^5^ College of Public Health, Medical and Veterinary Sciences, James Cook University, Townsville, Queensland, Australia

^6^Department of Zoology, Centre for Applied Genetics, Jnana Bharathi Campus, Bangalore University, Bengaluru, Karnataka, India

^7^Padmaja Naidu Himalayan Zoological Park, Darjeeling, India

^%^ Present address: Department of Biosciences, Chandigarh University, Mohali, Punjab, India

^#^Present address: Ashoka University, Rajiv Gandhi Education City, Sonipat, Haryana, India

*Corresponding author: [karthik@ccmb.res.in](mailto:karthik@ccmb.res.in)

**Supplementary Tables and Figures – Sreedharan et al.**

**Description:** It consists SRR files of 40 *Bd* chosen for alignment and reconstruction of ITS sequence; List of samples tested for *Bd* using the new primer; List of samples tested from Panama; Agarose gel image of the temperature gradient PCR; Image of the forward and reverse binding sites of ITS region of *Bd* isolates; Agarose gel image showing the amplicon at 82 bp of the positive control and additional band observed in some field samples; Graphs showing ∆Cq values which indicates the difference in the amplification cycles of Boyle’s and new primers; Graph showing the distribution of the reported ITS copy numbers between Boyle’s primers and new primers; and MIQE checklist

*Table S1:* *SRR files of 40 Bd isolates (NCBI BioProject accession PRJNA413876*) *chosen for alignment and reconstruction of ITS sequence, with the SRR and sample IDs, geographical information and identity of strain.*

| SRR ID | COUNTRY | CONTINENT | SAMPLE ID | STRAIN |
| --- | --- | --- | --- | --- |
| 6375480 | South Africa | Africa | Bd-GPL 08MG02 | GPL |
| 6375481 | South Africa | Africa | Bd-GPL 08MG05 | GPL |
| 6375506 | South Africa | Africa | SA-EC6 | GPL |
| 6375529 | South Africa | Africa | SA-KN4 | CAPE |
| 6375530 | South Africa | Africa | SAFS-1 | CAPE |
| 6375531 | South Africa | Africa | SA-KN1 | CAPE |
| 6375546 | South Africa | Africa | SA3e | CAPE |
| 6375547 | South Africa | Africa | MC58 | CAPE |
| 6375548 | South Africa | Africa | DB8-4 | CAPE |
| 6375588 | South Africa | Africa | SA-EC3 | Hybrid |
|  |  |  |  |  |
| 6375443 | Taiwan | Asia | TW16-304 | GPL |
| 6375468 | Taiwan | Asia | TW16-050 | GPL |
| 6375522 | South Korea | Asia | KB23 | ASIA-2 |
| 6375523 | South Korea | Asia | KB45 | ASIA-2 |
| 6375586 | South Korea | Asia | KB72 | ASIA-2 |
| 6375580 | South Korea | Asia | KRBOOR-323 | ASIA-1 |
| 6375581 | South Korea | Asia | KRBOOR-317 | ASIA-1 |
| 6375582 | South Korea | Asia | KBO-327 | ASIA-1 |
| 6375583 | South Korea | Asia | KBO-319 | ASIA-1 |
| 6375585 | South Korea | Asia | KB347 | ASIA-1 |

| SRR ID | COUNTRY | CONTINENT | SAMPLE ID | STRAIN |
| --- | --- | --- | --- | --- |
| 6378403 | France | Europe | ANS_13No1 | GPL |
| 6378420 | France | Europe | Lhurs_13No13 | GPL |
| 6375436 | Germany | Europe | BAV_BRUCH_1 | GPL |
| 6375491 | Hungary | Europe | Hung_2014 | GPL |
| 6375521 | Sweden | Europe | SWED-40-5-04 | GPL |
| 6375578 | Spain | Europe | Acherito_c_15 | GPL |
| 6375452 | Spain | Europe | CCB1 | CAPE |
| 6375544 | UK (Trade:WC Cameroon) | Europe | LM2 | CAPE |
| 6375549 | Mallorca | Europe | CCB15 | CAPE |
| 6375451 | Switzerland | Europe | 739 | CH |
|  |  |  |  |  |
| 6252317 | Chile | South America | AVS2 | GPL |
| 6252318 | Chile | South America | AVS7 | GPL |
| 6375463 | French Guinea | South America | FG32 | GPL |
| 6375524 | Brazil | South America | CLFT001 | ASIA-2 |
| 6375563 | Brazil | South America | CLFT061 | ASIA-2 |
| 6375564 | Brazil | South America | CLFT065 | ASIA-2 |
| 6375565 | Brazil | South America | CLFT067 | ASIA-2 |
| 6375566 | Brazil | South America | CLFT144 | ASIA-2 |
| 6375567 | Brazil | South America | CLFT071 | ASIA-2 |
| 6375571 | Brazil | South America | CLFT136 | ASIA-2 |

*Table S2:* *All the samples tested for Bd with the SYBR-Green based assay using the new primer set including anuran samples from 2014-2021, Caecilian samples from Bangalore University and Salamander samples from Darjeeling.*

| 2014-2016 | | | |
| --- | --- | --- | --- |
| Family | **Species** | **No: of positive \| total tested (95% CI prevalence)** | **Avg. Infection load ± SD** |
| Dicroglossidae | *Sphaerotheca dobsonni* | 8\|16 (28-72) | 718±537.5 |
|  | *Euphlyctis cyanophlyctis* | 12\|22(35-73) | 684±539.7 |
|  | *Minervarya caperata* | 18\|29(44-77) | 804±529.2 |
|  | *Hoplobatrachus tigerinus* | 41\|66(50-73) | 901±521.2 |
| Ranidae | *Indosylvirana caeseri* | 0\|1 | nil |
| Microhylidae | *Microhyla ornata* | 1\|1 | 576 |
| Rhacophoridae | *Polypedatus macaulatus* | 1\|1 | 1810 |
| Bufonidae | *Duttaphrynus melanostictus* | 1\|1 | 646 |
| 2018-2021 | | | |
| Dicroglossidae | *Euphlyctis cyanophlyctis* | 29\|32(75-98) | 4620±2710.3 |
|  | *Minervarya caperata* | 4\|4 | 3060±2920 |
|  | *Hoplobatrachus tigerinus* | 1\|1 | 3620 |
| Bufonidae | *Duttaphrynis melanostictus* | 1\|2 (9-91) | 7430 |
|  | *Pedostibes tuberculosus* | 0\|1 | nil |
| Ranidae | *Indosylvirana caeseri* | 5\|7(35-92) | 8280±20403 |
| Ranixalidae | *Indirana chirawasi* | 31\|33 (79-99) | 11987±21502 |
| Nyctibtrachidae | *Nyctibatrachus jog* | 6\|7(47-99) | 11400±7970 |
| Rhacophoridae | *Polypedatus macaulatus* | 2\|3(20-94) | 4300±2256.3 |
| Caecilians (2021) | | | |
| Grandisoniidae | *Gegeneophis orientalis* | 8\|17(26-69) | 839±480.7 |
|  | *Gegeneophis sp.nov.* | 7\|9(44-95) | 2760±5283 |
| Ichthyophiidae | *Ichthyophis beddomei* | 13\|14 (66-99) | 1000±595.9 |
|  | *Ichthyophis* | 7\|8(51-99) | 966±557.4 |
|  | *Ichthyophis kodaguensis* | 2\|2 | 908±262.5 |
|  | *Ichthyophis.cf. kodaguensis* | 1\|1 | 1610 |
|  | *Uraeotyphlus narayani* | 1\|1 | 561 |
|  | *Ichthyophis longicephalus* | 1\|1 | 663 |
|  | *Ichthyophis.cf. longicephalus* | 4\|4 | 1625±607 |
| Salamander (2020) | | | |
| Salamandridae | *Tylototriton verrucosus* | 11\|11 | 14600±10785.7 |

*Table S3:* *All samples tested at Panama, with species ID, infection status of the tested sample, and the mean Cq and concentration of the Bd ZE in the sample.*

|  | New Primers | | | Boyle's Primers | | | |  |
| --- | --- | --- | --- | --- | --- | --- | --- | --- |
| Species | Infection Status | Cq Mean | Mean concentration | | Infection Status | Cq Mean | Mean concentration | |
| *Sachatamia albomaculata* | - | 0.0 | 0.00E+00 | | - | 0.0 | 0.00E+00 | |
| *Atelopus limosus* | - | 0.0 | 0.00E+00 | | - | 0.0 | 0.00E+00 | |
| *Sachatamia albomaculata* | - | 0.0 | 0.00E+00 | | - | 0.0 | 0.00E+00 | |
| *Pristimantis sp.* | - | 0.0 | 0.00E+00 | | - | 0.0 | 0.00E+00 | |
| *Atelopus limosus* | - | 0.0 | 0.00E+00 | | - | 0.0 | 0.00E+00 | |
| *Rhaebo haematiticus* | - | 0.0 | 0.00E+00 | | - | 0.0 | 0.00E+00 | |
| *Rhaebo haematiticus* | - | 0.0 | 0.00E+00 | | - | 0.0 | 0.00E+00 | |
| *Teratohyla spinosa* | - | 0.0 | 0.00E+00 | | - | 0.0 | 0.00E+00 | |
| *Atelopus limosus* | - | 0.0 | 0.00E+00 | | - | 0.0 | 0.00E+00 | |
| *Atelopus limosus* | - | 0.0 | 0.00E+00 | | - | 0.0 | 0.00E+00 | |
| *Smilisca sila* | - | 0.0 | 0.00E+00 | | - | 0.0 | 0.00E+00 | |
| *Silverstoneia flotator* | - | 0.0 | 0.00E+00 | | - | 0.0 | 0.00E+00 | |
| *Rhaebo haematiticus* | - | 0.0 | 0.00E+00 | | - | 0.0 | 0.00E+00 | |
| *Colostethus pratti* | - | 0.0 | 0.00E+00 | | - | 0.0 | 0.00E+00 | |
| *Atelopus varius* | + | 20.9 | 1.67E+06 | | + | 23.5 | 9.01E+04 | |
| *Atelopus limosus* | + | 24.0 | 2.08E+05 | | + | 28.2 | 4.74E+03 | |
| *Atelopus varius* | + | 24.5 | 1.46E+05 | | + | 28.0 | 5.02E+03 | |
| *Atelopus limosus* | + | 26.6 | 2.90E+03 | | + | 26.6 | 1.29E+04 | |
| *Atelopus varius* | + | 27.4 | 1.69E+04 | | + | 30.5 | 1.82E+03 | |
| *Diasporus cf. diastema* | + | 28.1 | 1.65E+04 | | + | 27.0 | 9.98E+03 | |
| *Silverstoneia flotator* | + | 28.6 | 7.94E+03 | | + | 29.9 | 1.49E+03 | |
| *Atelopus limosus* | + | 29.1 | 3.03E+03 | | + | 29.7 | 1.81E+03 | |
| *Oophaga vicentei* | + | 29.3 | 4.27E+03 | | + | 32.1 | 8.02E+02 | |
| *Andinobates geminisae* | + | 30.1 | 2.37E+03 | | + | 30.8 | 1.23E+03 | |
| *Andinobates geminisae* | + | 30.7 | 1.61E+03 | | + | 31.1 | 9.90E+02 | |
| *Atelopus limosus* | + | 31.0 | 3.00E+03 | | + | 31.5 | 5.11E+02 | |
| *Craugastor evanesco* | + | 31.3 | 3.33E+03 | | + | 34.1 | 2.28E+02 | |
| *Dendrobates auratus* | + | 32.1 | 6.93E+02 | | + | 33.1 | 2.78E+02 | |
| *Teratohyla spinosa* | + | 32.9 | 3.41E+02 | | + | 34.0 | 1.00E+02 | |
| *Craugastor crassidigitus* | + | 32.9 | 2.94E+02 | | + | 34.6 | 1.27E+02 | |
| *Pristimantis cruentus* | + | 33.5 | 2.23E+02 | | + | 35.7 | 9.29E+01 | |
| *Atelopus varius* | + | 33.7 | 3.43E+03 | | + | 37.9 | 2.54E+01 | |
| *Rhaebo haematiticus* | + | 34.0 | 1.94E+03 | | + | 38.4 | 1.92E+01 | |
| *Hyalinobatrachium colymbiphylum* | + | 34.2 | 1.05E+02 | | + | 34.5 | 6.94E+01 | |
| *Colostethus sp.* | + | 34.3 | 1.19E+02 | | + | 35.3 | 4.80E+01 | |
| *Craugastor longirostris* | + | 34.4 | 8.84E+01 | | + | 37.3 | 3.68E+01 | |
| *Craugastor fitzingeri* | + | 34.4 | 8.61E+01 | | + | 33.6 | 1.32E+02 | |
| *Atelopus varius* | + | 35.0 | 3.44E+03 | | + | 41.8 | 3.98E+00 | |
| *Colostethus panamensis* | + | 35.0 | 2.96E+03 | | + | 35.7 | 3.26E+01 | |
| *Colostethus cf. pratti* | + | 35.0 | 3.16E+01 | | + | 36.3 | 2.29E+01 | |
| *Allobates talamancae* | + | 35.0 | 3.16E+01 | | + | 36.2 | 2.96E+01 | |
| *Diasporus diastema* | + | 35.0 | 3.16E+01 | | + | 35.1 | 1.32E+02 | |
| *Andinobates geminisae* | + | 35.0 | 3.16E+01 | | + | 36.4 | 4.69E+01 | |

*Fig S1:* *The agarose gel picture of a temperature gradient PCR done with the positive control (JEL197) as template. The temperature and the dilution factor of the template is marked.*


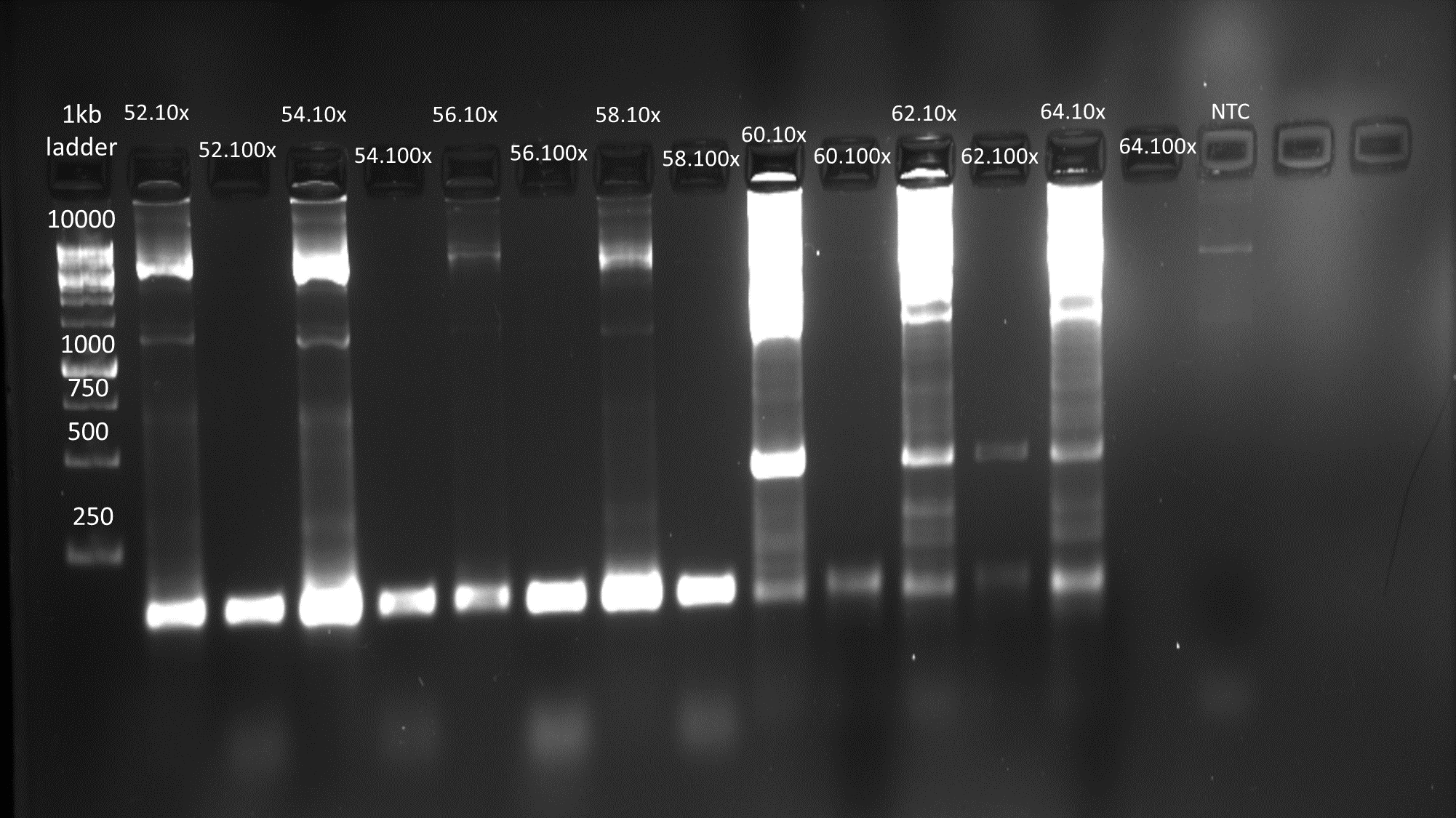


*Fig S2:* *Figure shows the forward and reverse primer binding sequences (shown in red) within the ITS for all the 40 sequences aligned from the raw short read files, covering all the lineages and continents.*

**SRR ID** **ALIGNED ITS SEQUENCE (from base 295 to 386 of the reference)** **STRAIN**

AFRICA

6375480 295 TTGAACGCACATTGCACTCGTAAAAGAGTATACATGTTTGAGAATTATAAAAATACATTGTCCGAATTGACTGGACAGATATGAACCATGCC 386 GPL

6375481 295 TTGAACGCACATTGCACTCGTAAAAGAGTATACATGTTTGAGAATTATAAAAATACATTGTCCGAATTGACTGGACAGATATGAACCATGTC 386 GPL

6375506 295 TTGAACGCACATTGCACTCGTAAAAGAGTATACATGTTTGAGAATTATAAAAATACATTGTCCGAATTGACTGGACAGATATGAACCATGTC 386 GPL

6375529 295 TTGAACGCACATTGCACTCGTAAAAGAGTATACATGTTTGAGAATTATAAAAATACATTGTCCGAATTGACTGGACAGATATGAACCATGTC 386 CAPE

6375530 295 TTGAACGCACATTGCACTCGTAAAAGAGTATACATGTTTGAGAATTATAAAAATACATTGTCCGAATTGACTGGACAGATATGAACCATGTC 386 CAPE

6375531 295 TTGAACGCACATTGCACTCGTAAAAGAGTATACATGTTTGAGAATTATAAAAATACATTGTCCGAATTGACTGGACAGATATGAACCATGTC 386 CAPE

6375546 295 TTGAACGCACATTGCACTCGTAAAAGAGTATACATGTTTGAGAATTATAAAAATACATTGTCCGAATTGACTGGACAGATATGAACCATGTC 386 CAPE

6375547 295 TTGAACGCACATTGCACTCGTAAAAGAGTATACATGTTTGAGAATTATAAAAATACATTGTCCGAATTGACTGGACAGATATGAACCATGTC 386 CAPE

6375548 295 TTGAACGCACATTGCACTCGTAAAAGAGTATACATGTTTGAGAATTATAAAAATACATTGTCCGAATTGACTGGACAGATATGAACCATGTC 386 CAPE

6375588 295 TTGAACGCACATTGCACTCGTAAAAGAGTATACATGTTTGAGAATTATAAAAATACATTGTCCGAATTGACTGGACAGATATGAACCATGTC 386 HYBRID

ASIA

6375443 295 TTGAACGCACATTGCACTCGTAAAAGAGTATACATGTTTGAGAATTATAAAAATACATTGTCCGAATTGACTGGACAGATATGAACCATGTC 386 GPL

6375468 295 TTGAACGCACATTGCACTCGTAAAAGAGTATACATGTTTGAGAATTATAAAAATACATTGTCCGAATTGACTGGACAGATATGAACCATGTC 386 GPL

6375522 295 TTGAACGCACATTGCACTCGTAAAAGAGTATACATGTTTGAGAATTATAAAAATACATTGTCCGAATTGACTGGACAGATATGAACCATGTC 386 ASIA-2

6375523 295 TTGAACGCACATTGCACTCGTAAAAGAGTATACATGTTTGAGAATTATAAAAATACATTGTCCGAATTGACTGGACAGATATGAACCATGTC 386 ASIA-2

6375586 295 TTGAACGCACATTGCACTCGTAAAAGAGTATACATGTTTGAGAATTATAAAAATACATTGTCCGAATTGACTGGACAGATATGAACCATGTC 386 ASIA-2

6375580 295 TTGAACGCACATTGCACTCGTAAAAGAGTATACATGTTTGAGAATTATAAAAATACATTGTCCGAATTGACTGGACAGATATGAACCATGTC 386 ASIA-1

6375581 295 TTGAACGCACATTGCACTCGTAAAAGAGTATACATGTTTGAGAATTATAAAAATACATTGTCCGAATTGACTGGACAGATATGAACCATGTC 386 ASIA-1

6375582 295 TTGAACGCACATTGCACTCGTAAAAGAGTATACATGTTTGAGAATTATAAAAATACATTGTCCGAATTGACTGGACAGATATGAACCATGTC 386 ASIA-1

6375583 295 TTGAACGCACATTGCACTCGTAAAAGAGTATACATGTTTGAGAATTATAAAAATACATTGTCCGAATTGACTGGACAGATATGAACCATGTC 386 ASIA-1

6375585 295 TTGAACGCACATTGCACTCGTAAAAGAGTATACATGTTTGAGAATTATAAAAATACATTGTCCGAATTGACTGGACAGATATGAACCATGTC 386 ASIA-1

EUROPE

6378403 295 TTGAACGCACATTGCACTCGTAAAAGAGTATACATGTTTGAGAATTATAAAAATACATTGTCCGAATTGACTGGACAGATATGAACCATGTC 386 GPL

6378420 295 TTGAACGCACATTGCACTCGTAAAAGAGTATACATGTTTGAGAATTATAAAAATACATTGTCCGAATTGACTGGACAGATATGAACCATGCC 386 GPL

6375436 295 TTGAACGCACATTGCACTCGTAAAAGAGTATACATGTTTGAGAATTATAAAAATACATTGTCCGAATTGACTGGACAGATATGAACCATGTC 386 GPL

6375491 295 TTGAACGCACATTGCACTCGTAAAAGAGTATACATGTTTGAGAATTATAAAAATACATTGTCCGAATTGACTGGACAGATATGAACCATGTC 386 GPL

6375521 295 TTGAACGCACATTGCACTCGTAAAAGAGTATACATGTTTGAGAATTATAAAAATACATTGTCCGAATTGACTGGACAGATATGAACCATGTC 386 GPL

6375578 295 TTGAACGCACATTGCACTCGTAAAAGAGTATACATGTTTGAGAATTATAAAAATACATTGTCCGAATTGACTGGACAGATATGAACCATGCC 386 GPL

6375452 295 TTGAACGCACATTGCACTCGTAAAAGAGTATACATGTTTGAGAATTATAAAAATACATTGTCCGAATTGACTGGACAGATATGAACCATGTC 386 GPL

6375544 295 TTGAACGCACATTGCACTCGTAAAAGAGTATACATGTTTGAGAATTATAAAAATACATTGTCCGAATTGACTGGACAGATATGAACCATGTC 386 CAPE

6375549 295 TTGAACGCACATTGCACTCGTAAAAGAGTATACATGTTTGAGAATTATAAAAATACATTGTCCGAATTGACTGGACAGATATGAACCATGTC 386 CAPE

6375451 295 TTGAACGCACATTGCACTCGTAAAAGAGTATACATGTTTGAGAATTATAAAAATACATTGTCCGAATTGACTGGACAGATATGAACCATGTC 386 CH

SOUTH AMERICA

6252317 295 TTGAACGCACATTGCACTCGTAAAAGAGTATACATGTTTGAGAATTATAAAAATACATTGTCCGAATTGACTGGACAGATATGAACCATGTC 386 GPL

6252318 295 TTGAACGCACATTGCACTCGTAAAAGAGTATACATGTTTGAGAATTATAAAAATACATTGTCCGAATTGACTGGACAGATATGAACCATGCC 386 GPL

6375463 295 TTGAACGCACATTGCACTCGTAAAAGAGTATACATGTTTGAGAATTATAAAAATACATTGTCCGAATTGACTGGACAGATATGAACCATGTC 386 GPL

6375524 295 TTGAACGCACATTGCACTCGTAAAAGAGTATACATGTTTGAGAATTATAAAAATACATTGTCCGAATTGACTGGACAGATATGAACCATGTC 386 ASIA-2

6375563 295 TTGAACGCACATTGCACTCGTAAAAGAGTATACATGTTTGAGAATTATAAAAATACATTGTCCGAATTGACTGGACAGATATGAACCATGTC 386 ASIA-2

6375564 295 TTGAACGCACATTGCACTCGTAAAAGAGTATACATGTTTGAGAATTATAAAAATACATTGTCCGAATTGACTGGACAGATATGAACCATGTC 386 ASIA-2

6375565 295 TTGAACGCACATTGCACTCGTAAAAGAGTATACATGTTTGAGAATTATAAAAATACATTGTCCGAATTGACTGGACAGATATGAACCATGTC 386 ASIA-2

6375566 295 TTGAACGCACATTGCACTCGTAAAAGAGTATACATGTTTGAGAATTATAAAAATACATTGTCCGAATTGACTGGACAGATATGAACCATGTC 386 ASIA-2

6375567 295 TTGAACGCACATTGCACTCGTAAAAGAGTATACATGTTTGAGAATTATAAAAATACATTGTCCGAATTGACTGGACAGATATGAACCATGTC 386 ASIA-2

6375571 295 TTGAACGCACATTGCACTCGTAAAAGAGTATACATGTTTGAGAATTATAAAAATACATTGTCCGAATTGACTGGACAGATATGAACCATGCC 386 ASIA-2

*Fig S3:* *A) An agarose gel image showing the amplicon at 82 bp of the positive control. The field samples with a second band below the 82 bp amplicon is shown. B) The melt curve corresponding to the standards constructed with JEL197 at 77◦C. C) The melt curve plot in which some samples have an additional peak at 79-80◦C.*

**A**


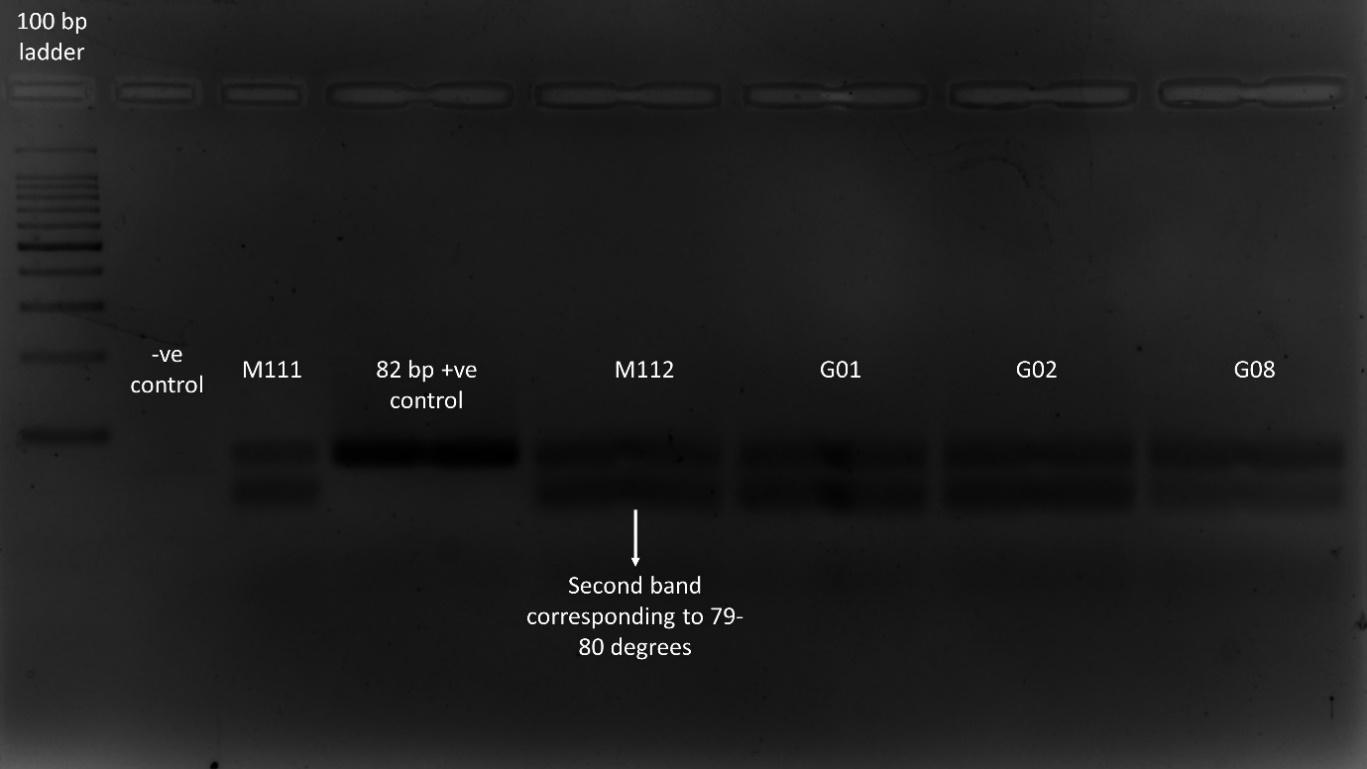


**B**


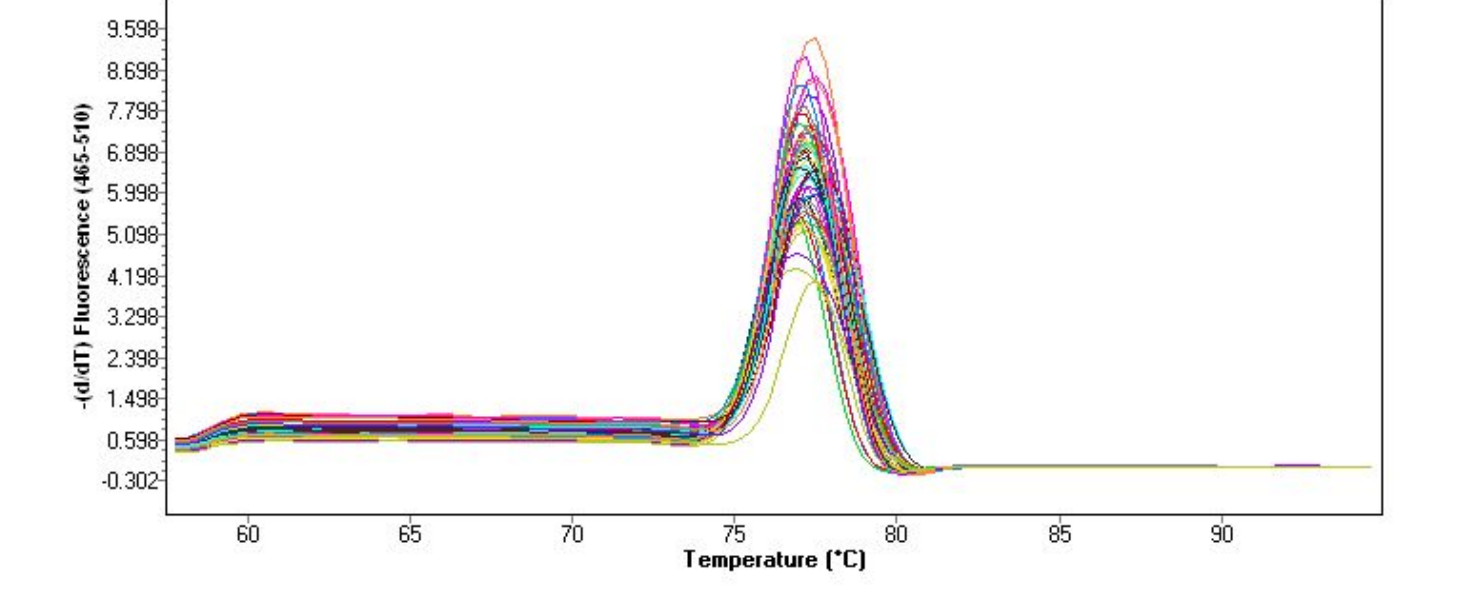


**C**


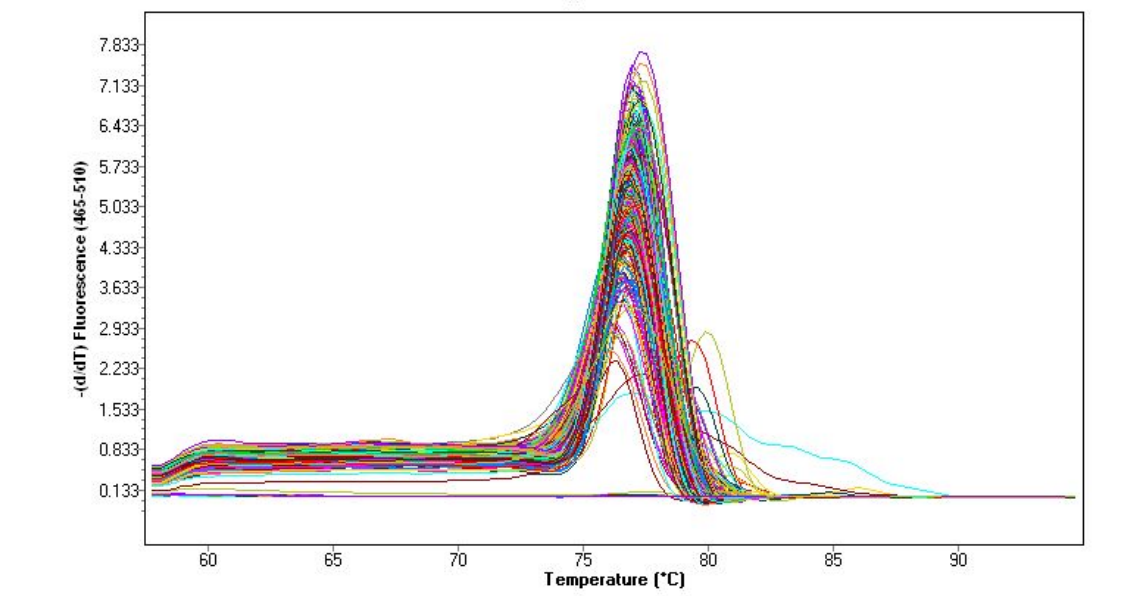


*Fig S4: A graph that shows the ∆Cq values and indicates the difference in the amplification cycles of Boyle’s and New primers.*

*
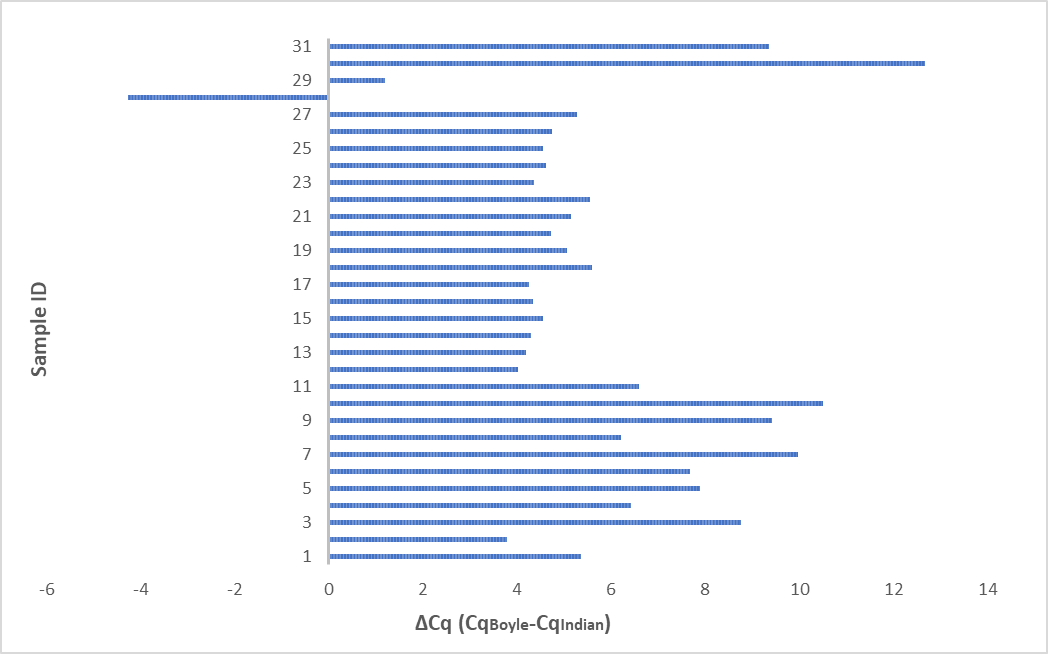
*

*Fig S5: A) A box plot showing the distribution of the reported ITS copy numbers between Boyle’s primers and new primers. This data compares the infection loads of samples (n=31) that are positive for both the methods. B) The Log_10_ Zoospore Equivalents reported by Boyle’s TaqMan based assay and the new SYBR Green based assay (n=30). C) Standard curve for the JEL423 with the new primers using the protocol. D) Standard curve for JEL423 with Boyles’ primers.*

*
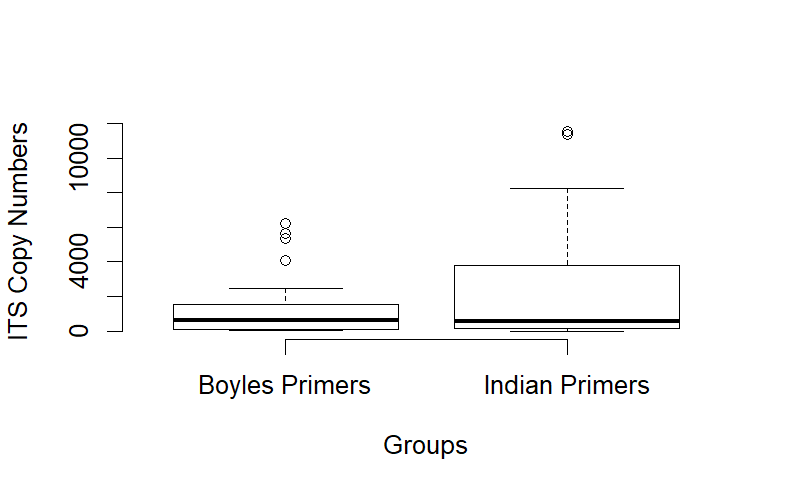
*

**A**


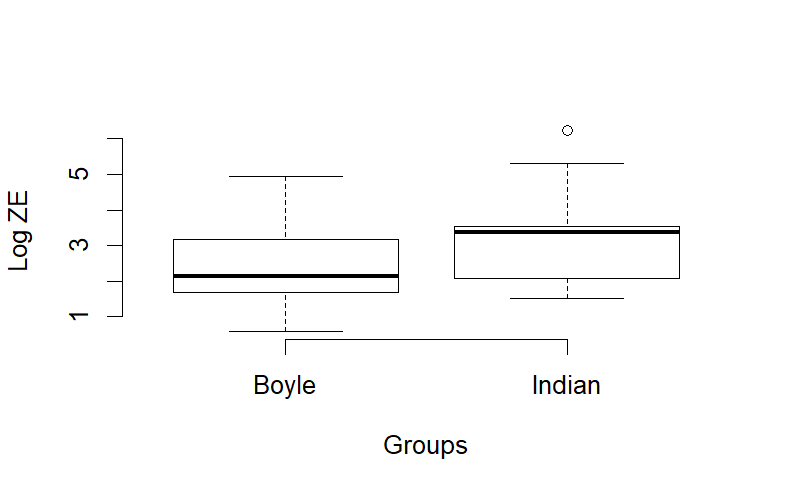


**B**

**C**


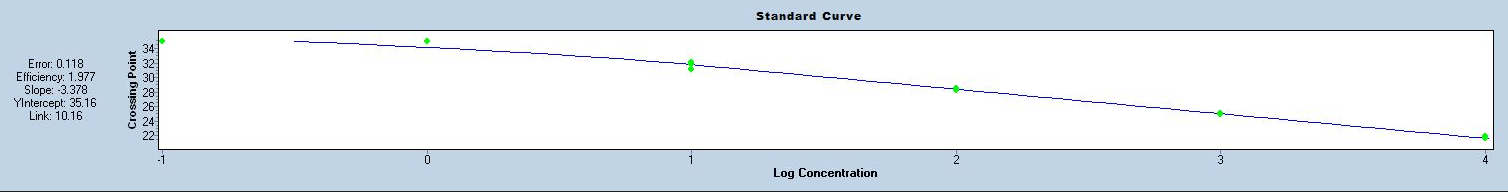


**D**


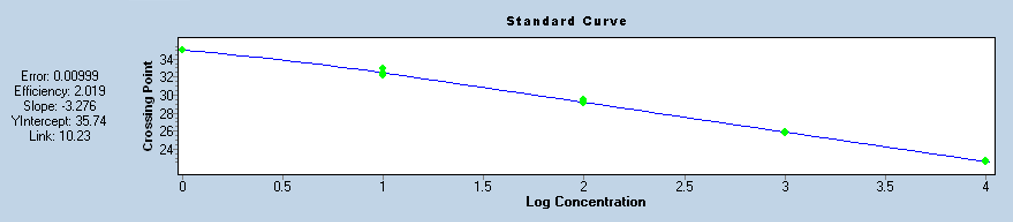


**Supplementary Information**

1. **Whole genome alignment and variant calling using bcftools and GATK**

For each of the forty SRR files, HISAT2 was used to first split the paired SRR files and convert them to fasta format using command *fastq-dump –split-files*. *hisat2-build* was used to index the reference genome, and *hisat2-x* used to align the reads to the reference. *samtools view* was used to convert the alignment file from sam format to bam format and sorted using *samtools sort* prior to variant calling. Bcftools *mpileup* was used for variant calling and consensus whole genome consensus sequences were built using bcftools *consensus*. BLAST against ITS reference was performed to get the consensus ITS sequence.

In the GATK variant calling pipeline (Kim et al., 2015), *samtools sort* and *samtools index* were used to sort and index the alignment file produced by HISAT2. GATK Picard *MarkDuplicates* were used to mark duplicates, and the output is again sorted and indexed using samtools. The reference fasta is indexed and dictionary created using *samtools faidx* and Picard *createdictionarysequence*. Finally, GATK *Haplotypecaller* was used to call variants, and bcftools *consensus* used to create the whole genome consensus sequence. All commands were applied using default parameters and all the alignments had sufficient read depth and per base coverage.

**MIQE Checklist**

| ITEM TO CHECK | IMPORTANCE | CHECKLIST |
| --- | --- | --- |
| EXPERIMENTAL DESIGN |  |  |
| Definition of experimental and control groups | **E** | Yes |
| Number within each group | **E** | Yes |
| Assay carried out by core lab or investigator's lab? | D | Core |
| Acknowledgement of authors' contributions | D | Yes |
| SAMPLE |  |  |
| Description | **E** | Yes |
| Volume/mass of sample processed | D | Yes |
| Microdissection or macrodissection | **E** | NA |
| Processing procedure | **E** | Yes |
| If frozen - how and how quickly? | **E** | Yes |
| If fixed - with what, how quickly? | **E** | NA |
| Sample storage conditions and duration (especially for FFPE samples) | **E** | NA |
| NUCLEIC ACID EXTRACTION |  |  |
| Procedure and/or instrumentation | **E** | Yes |
| Name of kit and details of any modifications | **E** | Yes |
| Source of additional reagents used | D |  |
| Details of DNase or RNAse treatment | **E** | NA |
| Contamination assessment (DNA or RNA) | **E** | Yes |
| Nucleic acid quantification | **E** | Yes |
| Instrument and method | **E** | Yes |
| Purity (A260/A280) | D |  |
| Yield | D |  |
| RNA integrity method/instrument | **E** | NA |
| RIN/RQI or Cq of 3' and 5' transcripts | **E** | NA |
| Electrophoresis traces | D | NA |
| Inhibition testing (Cq dilutions, spike or other) | **E** | Yes |
| REVERSE TRANSCRIPTION |  |  |
| Complete reaction conditions | **E** | Yes |
| Amount of RNA and reaction volume | **E** | Yes |
| Priming oligonucleotide (if using GSP) and concentration | **E** | Yes |
| Reverse transcriptase and concentration | **E** | NA |
| Temperature and time | **E** | Yes |
| Manufacturer of reagents and catalogue numbers | D | Yes |
| Cqs with and without RT | D* | NA |
| Storage conditions of cDNA | D | NA |
| qPCR TARGET INFORMATION |  |  |
| If multiplex, efficiency and LOD of each assay. | **E** | NA |
| Sequence accession number | **E** | NA |
| Location of amplicon | D | Yes |
| Amplicon length | **E** | Yes |
| *In silico* specificity screen (BLAST, etc) | **E** | Yes |
| Pseudogenes, retropseudogenes or other homologs? | D | NA |
| Sequence alignment | D | Yes |
| Secondary structure analysis of amplicon | D | Yes |
| Location of each primer by exon or intron (if applicable) | **E** | NA |
| What splice variants are targeted? | **E** | NA |
| qPCR OLIGONUCLEOTIDES |  |  |
| Primer sequences | **E** | Yes |
| RTPrimerDB Identification Number | D |  |
| Probe sequences | D** | NA |
| Location and identity of any modifications | **E** | NA |
| Manufacturer of oligonucleotides | D |  |
| Purification method | D |  |
| qPCR PROTOCOL |  |  |
| Complete reaction conditions | **E** | Yes |
| Reaction volume and amount of cDNA/DNA | **E** | Yes |
| Primer, (probe), Mg++ and dNTP concentrations | **E** | Yes |
| Polymerase identity and concentration | **E** | Yes |
| Buffer/kit identity and manufacturer | **E** | Yes |
| Exact chemical constitution of the buffer | D | Yes |
| Additives (SYBR Green I, DMSO, etc.) | **E** | Yes |
| Manufacturer of plates/tubes and catalog number | D |  |
| Complete thermocycling parameters | **E** | Yes |
| Reaction setup (manual/robotic) | D | Yes |
| Manufacturer of qPCR instrument | **E** | Yes |
| qPCR VALIDATION |  |  |
| Evidence of optimisation (from gradients) | D | Yes |
| Specificity (gel, sequence, melt, or digest) | **E** | Yes |
| For SYBR Green I, Cq of the NTC | **E** | Yes |
| Standard curves with slope and y-intercept | **E** | Yes |
| PCR efficiency calculated from slope | **E** | Yes |
| Confidence interval for PCR efficiency or standard error | D |  |
| r2 of standard curve | **E** | Yes |
| Linear dynamic range | **E** | Yes |
| Cq variation at lower limit | **E** | Yes |
| Confidence intervals throughout range | D |  |
| Evidence for limit of detection | **E** | Yes |
| If multiplex, efficiency and LOD of each assay. | **E** | NA |
| DATA ANALYSIS |  |  |
| qPCR analysis program (source, version) | **E** | R and Excel |
| Cq method determination | **E** | Second Derivative |
| Outlier identification and disposition | **E** | Yes |
| Results of NTCs | **E** | Yes |
| Justification of number and choice of reference genes | **E** | NA |
| Description of normalisation method | **E** | NA |
| Number and concordance of biological replicates | D |  |
| Number and stage (RT or qPCR) of technical replicates | **E** | Yes |
| Repeatability (intra-assay variation) | E |  |
| Reproducibility (inter-assay variation, %CV) | D |  |
| Power analysis | D |  |
| Statistical methods for result significance | **E** | Yes |
| Software (source, version) | E |  |
| Cq or raw data submission using RDML | **D** |  |
